# Feeding Assistance Skill Score: development and verification of reliability and validity

**DOI:** 10.1007/s41999-024-01016-8

**Published:** 2024-07-15

**Authors:** Ayano Nagano, Keisuke Maeda, Tomohiro Matsumoto, Kenta Murotani, Hidetaka Wakabayashi, Tamami Koyama, Takako Nagai, Naoharu Mori

**Affiliations:** 1 Department of Nursing, Nishinomiya Kyoritsu Neurosurgical Hospital, Nishinomiya, Hyogo Japan; 2 Palliative and Supportive Medicine, Graduate School of Medicine, Aichi Medical University, Nagakute, Aichi Japan; 3 Nutrition Therapy Support Center, Aichi Medical University Hospital, 1-1 Yazakokarimata, Nagakute, Aichi 480-1195 Japan; 4 Geriatric Medicine Hospital, National Center for Geriatrics and Gerontology, Obu, Aichi Japan; 5 General Medicine, Nerima-Hikarigaoka Hospital, Tokyo, Japan; 6 Biostatistics Center, Kurume University, Kurume, Fukuoka Japan; 7 School of Medical Technology, Kurume University, Kurume, Fukuoka Japan; 8 Department of Rehabilitation Medicine, Tokyo Women’s Medical University Hospital, Tokyo, Japan; 9 The Non-Profit Organization Kuchikara Taberu Shiawase-wo Mamoru-kai, Kanagawa, Japan

**Keywords:** Mealtime, Geriatrics, Nursing, Education

## Abstract

**Purpose:**

Feeding assistance should be safe and improve the assisted individual’s ability to feed, yet objective tools for evaluating these skills are lacking. This study focuses on developing a tool for assessing feeding assistance skills.

**Methods:**

A group consisting of 25 experts employed the Delphi method to achieve a consensus on the essential items necessary for assessing feeding assistance skills. Subsequently, a draft score using a three-point scale was developed. To test the reliability of the draft scores, a group of 20 patients and 20 nurses was matched to record a meal assistance scene, which were independently evaluated by three raters. We computed the AC_1_ statistic to assess both intra- and inter-rater reliability, and further examined correlations between the Feeding Assistance Skill Score (FASS) scores and outcome items to verify validity.

**Results:**

Initially, an 18-item draft score was generated using the Delphi method. Subsequently, seven items were omitted from the intra- or inter-rater reliability analysis. Furthermore, after discussion, researchers removed one item that assessed food cognition, because the agreement score between the two items was 91%. Finally, the 10-item FASS was developed, showing a correlation with dietary intake upon validation.

**Conclusions:**

We successfully developed a scoring system for peer evaluation of feeding assistance skills. Future studies should aim to validate the FASS. The implementation of the FASS aims to assess the effectiveness of feeding assistance training and care quality, potentially improving safety and satisfaction for those receiving feeding assistance.

**Supplementary Information:**

The online version contains supplementary material available at 10.1007/s41999-024-01016-8.

## Key summary points

**Aim:**

This study focuses on developing a tool for assessing feeding assistance skills.

**Findings:**

The 10-item Feeding Assistance Skill Score (FASS) was developed, showing a correlation with dietary intake upon validation.

**Message:**

FASS aims to assess the effectiveness of feeding assistance training and care quality, potentially improving safety and satisfaction for those receiving feeding assistance.

**Supplementary Information:**

The online version contains supplementary material available at 10.1007/s41999-024-01016-8.

## Introduction

Eating is a fundamental part of life, significantly affecting one’s Quality of Life (QOL), including the enjoyment of meals. Unfortunately, due to various factors such as dysphagia, upper limb paralysis, and cognitive decline, many people are unable to feed themselves and rely on feeding assistance. In hospital settings and institutionalized care, feeding assistance has proven beneficial, leading to improved outcomes such as increased food intake, weight gain, and enhanced nutritional status [1–5]. Additionally, research has reported that feeding assistance provided by trained caregivers enhances safety and increases patient satisfaction [4, 6]. Conversely, inappropriate feeding assistance increases the risk of aspiration, potentially leading to reduced food intake and the onset of aspiration pneumonia [7]. Deterioration in nutritional status is associated with conditions like sarcopenia and frailty, leading to negative outcomes such as reduced activities of daily living (ADL) and deteriorating swallowing function [8, 9]. Moreover, guidelines for nutrition in older adults also recommend providing mealtime assistance to increase food intake [10]. Thus, feeding assistance is crucial for maintaining the food intake and nutritional status of older people requiring care.

Feeding assistance is a daily living aid provided not only by formal caregivers such as nurses, but also by informal caregivers such as family caregivers and volunteers. According to a survey by the Japanese Ministry of Health, Labour and Welfare [11], approximately 40% of older people requiring nursing care reported needing assistance with meals. It has also been reported that care related to eating is associated with caregiving burden, along with excretion and changing clothes [12–14]. It was also reported that approximately 51.2% of all primary caregivers, regardless of whether they had feeding and swallowing difficulties, felt fearful about meal care, and meal care affects not only the physical burden but also the psychological burden [15]. In home care settings, the establishment of a safe feeding culture is essential, especially given the challenges posed by the lack of specialized training [16].

Feeding assistance encompasses a range of essential techniques aimed at preventing aspiration pneumonia and maintaining oral intake. It extends beyond the mere act of transferring food to the mouth using eating utensils. Effective eating assistance involves a combination of various techniques, such as preparing an optimal eating environment, adjusting the patient’s posture, increasing food awareness, encouragement of self-eating by assisting spoon handling and tailoring providing bite sizes and eating pace to align with the patient’s swallowing ability and cognitive function [17–19]. In addition to safe eating, it is also important to enjoy eating. Enhancing the feeding experience for the person receiving assistance, ensuring comfort and the absence of choking or distress, can lead to increased food intake and overall satisfaction. Furthermore, when family members assisting the patient experience reduced fear and a diminished sense of mealtime burden, it can foster a sense of security and happiness. Recent publications have introduced several tools for assessing mealtime engagement in individuals with dementia. Among these, the 18-item Mealtime Engagement Scale (MES) stands out for its moderate psychometric quality, demonstrating preliminary evidence of reliability and validity [19]. Such tools offer valuable insights into the dynamics of mealtime interactions with people living with dementia. However, there remains a gap in the availability of instruments specifically designed to evaluate the quality of feeding assistance provided by caregivers [20]. Addressing this gap is essential for developing targeted interventions that enhance the eating experience and nutritional intake of individuals with dementia.

The aim of this study was to develop an objective scale for evaluating feeding assistance skills and to verify its reliability and validity. This scale targets not only formal caregivers (such as nurses and care workers) but also informal caregivers (such as family members and friends who are non-professional care providers). In doing so, it can improve the feeding assistance skills of a wide range of caregivers and enhance the quality of training. This improvement can contribute to the enhancement of the quality of life (QOL) and end-of-life care for the older people, promote collaboration among multidisciplinary teams in support of mealtime, and alleviate the physical and emotional burdens.

## Methods

This study consists of Phases 1–3. This study was approved by the Ethics Committee of the Nerima Hikarigaoka Hospital (ID: 2,151,701).

### Phase 1: collecting experts’ opinions

The Delphi method was used to consolidate the essential items required for feeding assistance skills. The Delphi method, originally developed by RAND Corporation, a US think tank [21], involves a process in which a group of experts anonymously complete questionnaires, followed by a statistical analysis of the responses being fed back to the group of experts, and a series of repetitions. The goal of this method is to narrow the range of responses through iterative questioning, striving for consensus among the panel of experts. In this study, we recruited 25 experts specializing in feeding assistance. These experts were recruited through the Japanese Society for Rehabilitation Nutrition mailing list, as well as the associated Facebook group, and the Japanese Society for Nursing Research on Eating and Swallowing Disorders mailing list. The target experts were physicians, nurses, speech-language pathologists, dietitians, and others who held national qualifications and expert qualifications in feeding assistance, such as certified nurses in dysphagia, Japanese Society of Dysphagia Rehabilitation certified instructors, Japanese Society of Parental and Enteral Nutrition Therapy certified professionals for nutrition support, and Japanese Association of Rehabilitation Nutrition certified instructors. Basic information about the expert group was collected using an anonymous questionnaire. The collected information included job title, years of experience in their current role, specific expert qualification in feeding assistance, number of years since obtaining these qualifications, and their participation in nutrition- or swallowing-related conferences.

First, the research team developed an initial draft score consisting of 30 items. This was informed by an extensive initial literature review, incorporating both domestic and international sources to identify key elements for a robust feeding assistance instrument. Our expert team critically assessed these items, ensuring that the draft was grounded in current best practices and scientific knowledge in feeding assistance. An anonymous questionnaire was distributed to the group of experts using Google Forms. Participants responded on a 5-point scale (5 being the highest agreement) regarding their level of agreement with the draft score as a whole. They were also asked to select items that should be excluded from the evaluation and state the reasons for their exclusion (regardless of the number of items selected).

The research team compiled the responses from the questionnaire and revised the draft scores accordingly. Subsequently, we conducted a second round of the same questionnaire survey on the revised draft score, gathering opinions to reach a consensus. We established the endpoint based on the fulfillment of one of the following criteria: either after ten rounds of questionnaire surveys and opinion consolidation, when 70% of the respondents reached an agreement at level 4 or higher, when consensus was reached regarding opinions for exclusion, or when strong disagreements were eliminated. The proposed score for the items on which consensus was reached was rated on a three-point scale of 0 (not done), 1 (not enough), and 2 (done).

### Phase 2: inter- and intra-reliability

Phase 2 is the process of confirming the reliability of the proposed score generated in Phase 1. -point scale of 0.1.2. To test inter-reliability, examiners independently scored nurses' feeding assistance skills using the proposed score, based on 20 videos of patients receiving assistance at mealtimes. In addition, to test intra-reliability, a second assessment was performed on three randomly selected videos one hour after the first assessment. These videos of feeding assistance were recorded at the Nerima Hikarigaoka Hospital. Feeding assistance was provided by a random combination of 20 patients and 20 nurses who consented to participate in the study and were filmed under the same conditions by the researcher (T.M.). A sample size of 19 achieves 80% power to detect a difference of 0.6 between the null hypothesis correlation of 0.0 and the alternative hypothesis correlation of 0.6 using a two-sided hypothesis test with a significance level of 0.05. Thus, we set *N* = 20 as the required number of cases for each patient and nurse, accounting for a potential dropout of one patient. The number of cases was determined using the Pearson's correlation test module of PASS 2019.

The eligibility criteria for patients requiring assistance were as follows: age 65 years or older who were hospitalized at Nerima Hikarigaoka Hospital, required feeding assistance, and whose oral intake in the last two days was less than 50% of the amount provided. This criterion aimed to avoid the ceiling effect that could obscure the effectiveness of feeding assistance interventions. Focusing on patients with lower intake allowed a clear evaluation of tool in enhancing meal consumption. Information on the basic patient attributes was collected from medical records, including age, sex, primary illness, history related to feeding and swallowing disorders, and oral food intake scale level. The eligibility criteria for nurses were staff members at the Nerima Hikarigaoka Hospital, and a wide range of nurses were included, from those with extensive to limited experience in feeding assistance. Participants provided written informed consent, having been fully informed of the study's aims, procedures, potential risks, and benefits. They voluntarily agreed to participate in the study.

The three examiners were non-employees of Nerima Hikarigaoka Hospital, including doctors, nurses certified in feeding and swallowing disorders, and speech-language pathologists who had professional qualifications in feeding assistance and were able to give lectures and practical skills on feeding assistance.

Inter- and intra-reliability was verified using the AC_1_ statistic, which is less susceptible to bias, because the data were categorical variables (0, 1, 2), and the results were highly biased. The AC1 statistic of Gwet [22], which is less susceptible to bias, was used as an indicator for inter- and intra-reliability, as items with an AC1 statistic of less than 0.4 were considered to have low reliability and were excluded. The agreement between items in the same assessment category was calculated for each examinee (e.g., the proportion of patients with identical ratings for items 1 and 2 out of the 19 patients), and the median value for the three examinees was also calculated. Items with a high degree of agreement were judged to have the same content and the researcher selected items that seemed more appropriate from the expert’s perspective. The final items selected made up the Feeding Assistance Skill Score (FASS).

### Phase 3: validation

The mean scores of the three examiners in the movie assessment were calculated based on the FASS determined in Phase 2. Correlations were determined between the FASS scores and outcome items. Outcomes for validation included food intake (at the end of the meal; the difference in total weight before and after the meal was calculated), scores on a knowledge test on feeding assistance (the test was developed by the research group using a selection of four-choice questions from the entrance examinations for the educational process for Certified Nurse in Dysphasia Nursing; administered after filming a feeding assistance video), years of nursing experience, and expert qualifications such as certified nurse in feeding and swallowing disorders. The following information was collected: existence and type of qualifications, participation in conferences related to nutrition and feeding and swallowing disorders (total number of times), number of patients assisted with meals per month, whether video fluorography (VF) or videoendoscopic evaluation of swallowing (VE) was performed or assisted, participation in hospital nutrition support team (NST) or swallowing teams, participation in hands-on seminars and practical skills seminars on feeding assistance (total number of times), and number of books on feeding assistance owned. The number of books published on feeding assistance was recorded.

### The process of developing English version of FASS

The Development and Translation Process of the English Version of FASS:

The FASS was developed through a meticulous process, beginning with its validation in Japanese. This involved assessing the reliability and validity of an initial 18-item draft. Upon finalizing the ten most relevant items based on this validation, we engaged a professional translation service to conduct a double back-translation. This rigorous translation process was aimed at ensuring the English version of the FASS accurately reflected the validated Japanese version, both in content and in context.

## Results

### Phase 1

**Fig. 1:**
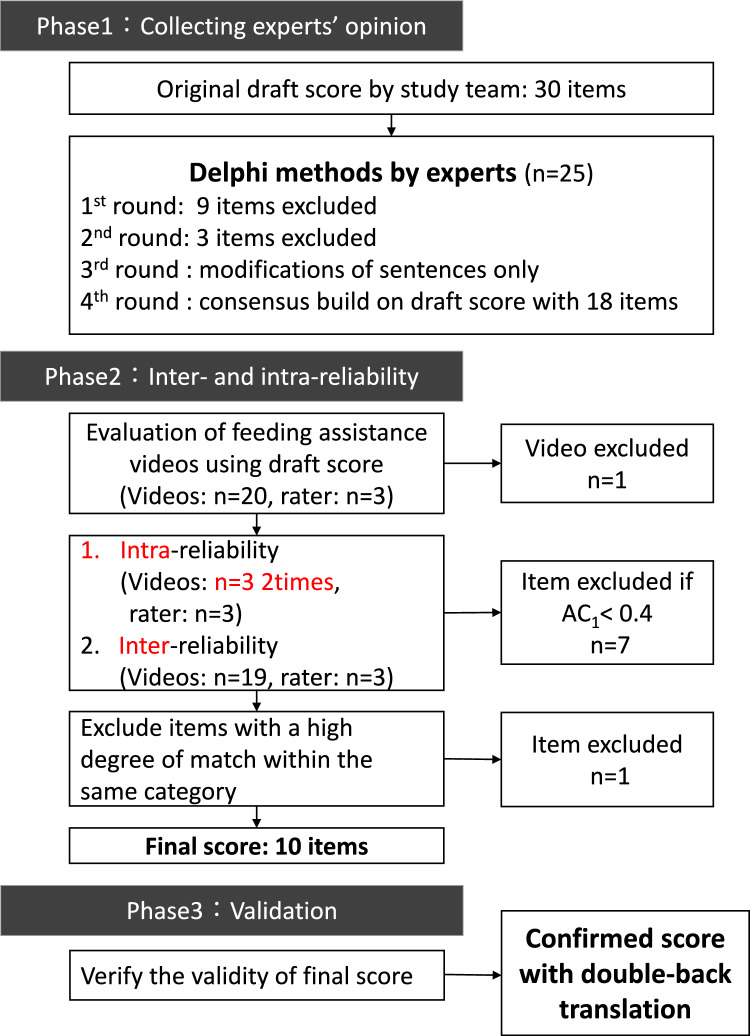
Study flow. This study consists three phases. Phase 1: A group consisting of 25 experts employed the Delphi method to achieve a consensus on the essential items necessary for assessing feeding assistance skills. Subsequently, a three-point scale draft score, based on the agreed-upon content, was developed. Phase 2: To test the reliability of the proposed scores, a group of 20 patients and 20 nurses were matched to record a meal assistance scene, which was independently evaluated by three raters using the proposed scores. We computed the AC1 statistic to assess both intra- and inter-rater reliability. Phase 3: Correlations between the FASS scores and outcome items were examined to verify validity

Twenty-five experts participated in the Delphi test. The basic demographic characteristics of the participants are presented in Supplementary Table 1. Four rounds were conducted to reach consensus on the final proposed score, which consisted of 18 items (Fig. 1). Opinions were collected from all 25 participants in all the rounds.

### Phase 2

Twenty feeding assistance videos were recorded. The basic patient characteristics are presented in Supplementary Table 2. Patients’ mean age was 88.2 ± 4.7 and 65% were female. The functional level of oral intake was the total oral diet of a single consistency, except for one patient who was tube-dependent with consistent oral intake of food or liquid. Three examiners independently evaluated the videos using the FASS; the three examiners comprised a doctor and two nurses. Nineteen videos in total were used for scoring, with one movie excluded, because it was too short for assessment. For inter- and intra-rater reliability, AC_1_ statistics were calculated for each item (Table 1). Eleven items remained after excluding items with AC_1_ statistics of less than 0.4. Next, agreement was calculated for items related to posture (items 1, 2, and 3), food recognition (items 9, 15, and 17), and spoon handling (items 11, 12, and 13). The experts judged that they evaluated the same content. The choice of which item to select was examined from the expert's point of view: item 11 'Manipulating the spoon so that the carer's jaw does not rise and inserting it into the mouth' observes a series of actions, such as scooping and then moving the spoon to the mouth. On the other hand, item 12 'Inserts the spoon so that the spoon hole is horizontal to the carer's tongue' only observes the action at the moment the spoon enters the mouth. Reliability was equal for both items; therefore, item 11, which had a larger observation range, was selected for analysis. Finally, a skill score consisting of ten items was determined (Table 2).

**Table 1:** Developmental process of the FASS: results of inter- and intra-reliability tests

	Items	Intra-reliability AC_1_	Inter-reliability AC_1_
1	Adjusting the care recipient’s body such that it does not lean to the right or left	0.440 [0.199, 0.681]	0.443 [− 0.332, 1]
2	Adjusting the care recipient’s feet such that the soles touch the floor, pedestal, etc	0.865 [0.692, 1]	1.000 [1, 1]
3	Adjusting the care recipient’s head and neck such that they are bent slightly forward	0.4855 [0.22, 0.749]	0.454 [− 0.133, 1]
4	The environment is conducive to concentrating on eating, e.g., by turning off the TV	0.194 [-0.072, 0.461]	0.544 [− 0.012, 1]
5	No non-food related items are placed on the table	0.318 [0.088, 0.548]	0.846 [0.488,1]
6	Explaining the menu to the extent that the care recipient can understand it	0.344 [0.088, 0.6]	1.000 [1, 1]
7	Making sure that there is no saliva or phlegm accumulation in the care recipient’s mouth before meals by, for example, requesting him or her to speak	0.883 [0.738, 1]	0.876 [0.558, 1]
8	Selecting spoons and other eating utensils suited to the feeding and swallowing abilities of the person being cared for	0.205 [0.088, 0.323]	1.000 [1, 1]
9	Scooping meals in a position visible to the care recipient	0.764 [0.572, 0.955]	0.876 [0.588,1]
10	When the caregiver provides assistance to the care recipient from the right side, the caregiver holds the spoon in the right hand, whereas when assistance is provided to the care recipient from the left side, the spoon is held in the left hand	0.955 [0.858, 1]	1.000 [1, 1]
11	Handling the spoon such that the care recipient does not need to raise his or her chin when it is put it in his or her mouth	0.501 [0.227, 0.776]	0.723 [0.21, 1]
12	Inserting the spoon so that the spoon hole is horizontal to the assisted Pearson’s tongue	0.620 [0.377, 0.864]	0.723 [0.21, 2]
13	Encouraging lip closure before the spoon is withdrawn	0.652 [0.415, 0.891]	0.289 [− 0.337, 0.914]
14	Ensuring that the spoon is scraped against the care recipient’s upper lip when it is removed	0.520 [0.286, 0.753]	0.788 [0.29, 1]
15	Being careful to ensure that the care recipient can see the food he or she is eating	0.602 [0.37, 0.834]	0.557 [− 0.016, 1]
16	Adjusting the appropriate bite size for the care recipient	0.163 [− 0.002, 0.329]	1.000 [1, 1]
17	Preparing the next morsel of food in a way that it does not disrupt the care recipient’s eating pace	0.517 [0.284, 0.75]	0.402 [− 0.197, 1]
18	No excessive talking to distract the care recipient before swallowing	0.193 [− 0.046, 0.431]	0.365 [− 0.421, 1]

**Table 2:** Final version of Feeding Assistance Skill Score following double back-translation

1	Adjusting the care recipient’s body such that it does not lean to the right or left
2	Adjusting the care recipient’s feet such that the soles touch the floor, pedestal, etc
3	Adjusting the care recipient’s head and neck such that they are bent slightly forward
4	Making sure that there is no saliva or phlegm accumulation in the care recipient’s mouth before meals by, for example, requesting him or her to speak
5	Scooping meals in a position visible to the care recipient
6	When the caregiver provides assistance to the care recipient from the right side, the caregiver holds the spoon in the right hand, whereas when assistance is provided to the care recipient from the left side, the spoon is held in the left hand
7	Handling the spoon such that the care recipient does not need to raise his or her chin when it is put it in his or her mouth
8	Ensuring that the spoon is scraped against the care recipient’s upper lip when it is removed
9	Being careful to ensure that the care recipient can see the food he or she is eating
10	Preparing the next morsel of food in a way that it does not disrupt the care recipient’s eating pace
	Each item is evaluated using a three-point scoring system: 0 indicates 'not done,' 1 indicates 'not done enough,' and 2 indicates 'done enough.' The Feeding Assistance Skill Score is calculated as the sum of these individual scores

### Phase 3

To test the validity of the study, we examined the basic attributes of the nurses and assessed the correlation between their dietary intake and FASS scores. Basic patient characteristics are presented in Supplementary Table 2. All participating nurses were female and had a median of 7 years of experience [median: 7.00, range: 4.50, 11.00]. None held expert qualifications, such as certification in dysphagia, or had attended relevant conferences. Additionally, none of the participants had participated in the VE/VF, NST, or swallowing teams. Out of the participants, seven (36.8%) attended feeding assistance training, while only one participant (5.3%) had a book about feeding assistance. We examined correlations between the mean FASS scores of the three examiners and various outcome items. The correlation between the years of nursing experience was minimal, with *R*^2^ = − 0.005, *p* = 0.825. The correlations between FASS scores and food intake, and nurses’ knowledge test scores were *R*^2^ = 0.318 (*p* = 0.006) and *R*^2^ = 0.120 (*p* = 0.103), respectively. No significant correlation was observed between the FASS scores and participation in in-hospital feeding assistance seminars *R*^2^ = 0.124 (*p* = 0.966).

The validation phase commenced with a proposed 18-item score created and assessed in Japanese during Phase 2. This phase's outcomes led to the refinement and finalization of a 10-item version of the FASS. Subsequently, this 10-item version was subject to a professional double back-translation, culminating in the production of the English version of the FASS (Table 2). This translation not only preserved the validated content but also made the instrument applicable for English-speaking contexts.

## Discussion

In this study, a tool for assessing others' feeding assistance skills was developed and tested for reliability and validity. A draft score of 18 items was developed using the Delphi method in a group of experts. The FASS, consisting of ten items, was completed through reliability testing, and weak correlation was found between the FASS score and food intake.

The FASS developed in the current study contained the items needed to assess feeding assistance skills. Feeding assistance requires appropriate eating environment, correct eating posture, and appropriate techniques to optimize oral intake and prevent aspiration [17–19]. The FASS included the following categories of feeding assistance techniques: eating posture, oral swallowing status, food perception, and spoon handling. Eating posture affects swallowing function [18, 23–25]. The neck extension position inhibits swallowing movements [25], whereas the chin tuck position effectively prevents aspiration [18]. Plantar grounding increased the efficiency of swallowing muscle activity [23]. A good eating posture also increases tongue pressure [26]. Older adults with disabilities or dementia have difficulty in adjusting and maintaining their posture by themselves and require postural adjustment by a carer [17, 27]. Checking the oral swallowing status confirms that the patient is capable of oral intake and helps select meal preparation and assistance methods suited to the swallowing function[28]. Creating an environment in which the patient can concentrate on eating and promoting food recognition is necessary to increase the awareness of swallowing, facilitate feeding and swallowing behavior [17], as well as to increase food intake [5]. Although evidence regarding spooning is lacking, spooning is required to maximize a patient's feeding ability. In addition, appropriate bolus adjustments are effective in preventing aspiration [18]. In this study, 25 clinically experienced experts in feeding assistance used the Delphi method to identify and consolidate the important factors in feeding assistance.

The reliability and validity of FASS as an objective tool for assessing feeding assistance skills was demonstrated, with reliability ensured by selecting items with an AC1 of 0.4 or more (moderate) in inter- and intra-reliability, and by eliminating duplication of assessment content. Validation showed a positive weak correlation between the FASS scores and feeding intake. Previous intervention studies have demonstrated that training in feeding assistance can lead to increased food intake, suggesting a link between improved feeding techniques/awareness and increased intake [3]. In our study, a weak correlation was found between FASS scores (reflecting the quality of feeding assistance techniques) and food intake. However, no significant correlation was observed between FASS scores and the number of years of nursing experience or attendance at feeding assistance seminars. This suggests that length of experience does not necessarily equate to the acquisition of appropriate feeding assistance techniques. Similarly, seminar attendance alone does not guarantee the acquisition of correct techniques, as the presence of hands-on training and the quality of seminars likely play crucial roles. We propose that the FASS, developed in this study, offers a novel approach to objectively assessing feeding assistance, paving the way for more effective training programs.

The strength of this study lies in the development of an index that can objectively assess caregivers’ skills. In general, many indicators evaluate the patient's perspective or subjective evaluation of the quality of nursing care [29–31], or evaluate the patient's challenges [32]. However, FASS is an indicator used by others to evaluate caregivers’ skills and can directly assess the quality of care and skills. This makes it possible to verify the effectiveness of feeding assistance training and the impact of quality of care on patient outcomes. One of the limitations of this study is that its validation was limited. The only outcome correlated with the FASS was dietary intake, demonstrating a weak correlation coefficient of 0.318. Furthermore, the study was confined to hospitalized older patients. Further validation using caregiver knowledge, experience, and feed intake in broader patients as outcomes is needed. An additional limitation of our study pertains to the incomplete evaluation of safety aspects in feeding assistance, particularly concerning safe swallowing and choking prevention. Although FASS developed through the Delphi process includes the item "Verification of clear vocalization before meal commencement to check for the absence of saliva or phlegm accumulation," it represents only a fraction of the safety measures initially considered. The initial draft, informed by a comprehensive literature review and expert consultation, encompassed multiple items related to ensuring safe swallowing. However, through the consensus-building phase, only the aforementioned item was retained. The prevention of choking and ensuring safe swallowing is paramount in feeding assistance, underscoring the necessity for the FASS to be validated against these critical safety outcomes in future studies. This effort will enhance its capacity to reduce feeding assistance risks and foster safer practices, broadening its use across various care settings. In addition, we acknowledge that the final English version of the FASS was produced through a double back-translation process but has not yet been validated in English. This underscores the need for future studies to validate the FASS in English-speaking populations to ensure its applicability and reliability across different linguistic contexts.

The FASS, which consists of ten items, was developed to enable objective assessment of feeding assistance skills, which may contribute to the development of research on feeding assistance and the improvement of feeding assistance skills in clinical practice. Assessment and education using the FASS target a wide range of people involved in meal care, including informal caregivers such as family caregivers, as well as formal care givers such as nurses. Support for eating is essential not only for older people in hospitals or those under institutional care but also in various settings such as palliative care at home. We hope that the development of the FASS will lead to the expansion of research on feeding assistance techniques and food support.

## Conclusion

In this study, a Feeding Assistance Skill Score was developed to objectively assess feeding assistance techniques, demonstrating reliability but limited validity in the studied outcomes. The FASS enables the assessment of feeding assistance skills by third parties which may contribute to the development of research on feeding assistance and improvement of feeding assistance skills in clinical practice.

## Supplementary Information

Below is the link to the electronic supplementary material.

Supplementary file1 (DOCX 15 KB)
