# Correction: Feeding Assistance Skill Score: development and verification of reliability and validity

**DOI:** 10.1007/s41999-024-01078-8

**Published:** 2024-11-22

**Authors:** Ayano Nagano, Keisuke Maeda, Tomohiro Matsumoto, Kenta Murotani, Hidetaka Wakabayashi, Tamami Koyama, Takako Nagai, Naoharu Mori

**Affiliations:** 1 Department of Nursing, Nishinomiya Kyoritsu Neurosurgical Hospital, Nishinomiya, Hyogo Japan; 2 Palliative and Supportive Medicine, Graduate School of Medicine, Aichi Medical University, Nagakute, Aichi Japan; 3 Nutrition Therapy Support Center, Aichi Medical University Hospital, 1-1 Yazakokarimata, Nagakute, Aichi 480-1195 Japan; 4 Geriatric Medicine Hospital, National Center for Geriatrics and Gerontology, Obu, Aichi Japan; 5 General Medicine, Nerima-Hikarigaoka Hospital, Tokyo, Japan; 6 Biostatistics Center, Kurume University, Kurume, Fukuoka Japan; 7 School of Medical Technology, Kurume University, Kurume, Fukuoka Japan; 8 Department of Rehabilitation Medicine, Tokyo Women’s Medical University Hospital, Tokyo, Japan; 9 The Non-Profit Organization Kuchikara Taberu Shiawase-wo Mamoru-kai, Kanagawa, Japan

**Correction: European Geriatric Medicine** 10.1007/s41999-024-01016-8

The article Feeding Assistance Skill Score: development and verification of reliability and validity, written by Maeda Keisuke, was originally published electronically on the publisher’s internet portal on 15.07.2024 without open access. With the author(s)’ decision to opt for Open Choice the copyright of the article changed on 07.07.2024 to © The Author(s), under exclusive licence to European Geriatric Medicine Society 2024 and the article is forthwith distributed under a Creative Commons Attribution.

