# Supplementary material for: Feeding Assistance Skill Score: development and verification of reliability and validity
Source: Eur Geriatr Med. 2024 Jul 15;15(5):1437–45. doi: 10.1007/s41999-024-01016-8 (PMC11614930; doi:10.1007/s41999-024-01016-8)
Supplement: Supplementary file 1 — Supplementary file1 (DOCX 15 KB) [file 41999_2024_1016_MOESM1_ESM.docx]

Supplemental Table 1: The characteristics of expert group

|  | **N** | **%** |
| --- | --- | --- |
| Profession |  |  |
| Doctor | 2 | 8.0 |
| Nurse | 9 | 36.0 |
| Speech and language therapist | 6 | 16.0 |
| Registered Dietitian | 4 | 24.0 |
| Dentist | 2 | 8.0 |
| Dental hygienist | 1 | 4.0 |
| Physiotherapist | 1 | 4.0 |
| years of experience |  |  |
| 10 years or over | 25 | 100 |
| Qualification |  |  |
| JSDR- certified instructor | 15 | 31.3 |
| JSPEN certificated professional for nutrition support | 12 | 25.0 |
| CN in dysphagia nursing | 6 | 12.5 |
| JARN-certified instructor | 6 | 12.5 |
| KTSM-certified instructor | 5 | 10.4 |
| Certified speech-language pathologist in swallowing | 3 | 6.3 |
| Board-certificated physiatrist | 1 | 20.8 |
| Years of qualified experience |  |  |
| < 5 years | 3 | 12.0 |
| ≥ 5 years and < 10 years | 9 | 36.0 |
| ≥ 10 years | 13 | 52.0 |
| Academic affiliations |  |  |
| JARN | 21 | 26.3 |
| JSDR | 20 | 25.0 |
| JSPEN | 17 | 21.3 |
| SSDJ | 9 | 11.3 |
| JSMCN | 8 | 10.0 |
| JNCM | 2 | 2.5 |
| Other | 3 | 13.6 |
| Experience of lectures | 20 | 80.0 |

JSDR, Japanese Society of Dysphagia Rehabilitation; JSPEN, Japanese Society of Clinical Nutrition and Metabolism; KTSM; Kushi-kara Taberu Shiawase-wo Mamorukai, CN; Certified nurse; JARN, Japanese Association of Rehabilitation Nutrition; SSDJ, Society of Swallowing and Dysphagia of Japan; JSMCN, Japan Society of Metabolism and Clinical Nutrition; JNCM, Japanese Society on Nutrition Care and Management

| Age, years | 88.2 ± 4.7 |
| --- | --- |
| Sex, female (%) | 13 (65.0) |
| Functional level of food intake (%) |  |
| Tube dependent with consistent oral intake of food or liquid | 1 ( 5.0) |
| Total oral diet of single consistency | 19 (95.0) |
| Hospitalization precipitating disease (%) |  |
| Respiratory infection | 7 ( 35.0) |
| Solid cancer | 5 (25.0) |
| Gastrointestinal/liver/pancreas disease | 3 ( 15.0) |
| Hematologic metabolic disorders | 4 (20.0) |
| Renal disease | 1 (5.0) |
| Dependency level, n (%) |  |
| Independent | 1 (5.0) |
| Moderately dependent | 11 (55.0) |
| Highly dependent | 8 (40.0) |

Supplemental Table 2. The characteristics of patients
